# Barriers and Facilitators to Heart Failure Guideline-Directed Medical Therapy in an Integrated Health System and Federally Qualified Health Centers: A Thematic Qualitative Analysis

**DOI:** 10.1007/s11606-025-09515-5

**Published:** 2025-05-05

**Authors:** Sarah E. Philbin, Lacey P. Gleason, Stephen D. Persell, Eve Walter, Lucia C. Petito, Anjan Tibrewala, Clyde W. Yancy, Rinad S. Beidas, Jane E. Wilcox, R. Kannan Mutharasan, Donald Lloyd-Jones, Matthew J. O’Brien, Abel N. Kho, Megan C. McHugh, Justin D. Smith, Faraz S. Ahmad

**Affiliations:** 1 Center for Education in Health Sciences, Northwestern University Feinberg School of Medicine, Chicago, IL USA; 2 Center for Health Information Partnerships, Institute for Public Health and Medicine, Northwestern University Feinberg School of Medicine, Chicago, IL USA; 3 Division of General Internal Medicine, Department of Medicine, Northwestern University Feinberg School of Medicine, Chicago, IL USA; 4 Center for Primary Care Innovation, Institute for Public Health and Medicine, Northwestern University Feinberg School of Medicine, Chicago, IL USA; 5 AllianceChicago, Chicago, IL USA; 6 Icahn School of Medicine at Mount Sinai, New York, NY USA; 7 Division of Biostatistics, Department of Preventive Medicine, Northwestern University Feinberg School of Medicine, Chicago, IL USA; 8 Division of Cardiology, Department of Medicine, Northwestern University Feinberg School of Medicine, Chicago, IL USA; 9 Department of Medical Social Sciences, Northwestern University Feinberg School of Medicine, Chicago, IL USA; 10 Department of Preventive Medicine, Northwestern University Feinberg School of Medicine, Chicago, IL USA; 11 Department of Emergency Medicine, Northwestern University Feinberg School of Medicine, Chicago, IL USA; 12 Center for Health Services and Outcomes Research, Institute for Public Health and Medicine, Northwestern University Feinberg School of Medicine, Chicago, IL USA; 13 Division of Health System Innovation and Research, Department of Population Health Sciences, Spencer Fox Eccles School of Medicine at the University of Utah, Salt Lake City, UT USA

**Keywords:** Heart failure, Quality of care, Qualitative methods, Implementation science, Determinants

## Abstract

**Background:**

Clinical guidelines recommend medications from four drug classes, collectively referred to as quadruple therapy, to improve outcomes for patients with heart failure with reduced ejection fraction (HFrEF). Wide gaps in uptake of these therapies persist across a range of settings. In this qualitative study, we identified determinants (i.e., barriers and facilitators) of quadruple therapy intensification, defined as prescribing a new class or increasing the dose of a currently prescribed medication.

**Methods:**

We conducted interviews with physicians, nurse practitioners, physician assistants, and pharmacists working in primary care or cardiology settings in an integrated health system or federally qualified health centers (FQHCs). We report results with a conceptual model integrating two frameworks: (1) the Theory of Planned Behavior (TPB), which explains how personal attitudes, perception of others’ attitudes, and perceived behavioral control influence intentions and behaviors; and (2) the Consolidated Framework for Implementation Research (CFIR) 2.0 to understand how multi-level factors influence attitudes toward and intention to use quadruple therapy.

**Results:**

Thirty-one clinicians, including 18 (58%) primary care and 13 (42%) cardiology clinicians, participated in the interviews. Eight (26%) participants were from FQHCs. A common facilitator in both settings was the belief in the importance of quadruple therapy. Common barriers included challenges presented by patient frailty, clinical inertia, and time constraints. In FQHCs, primary care comfort and ownership enhanced the intensification of quadruple therapy while limited access to and communication with cardiology specialists presented a barrier. Results are presented using a combined TPB-CFIR framework to help illustrate the potential impact of contextual factors on individual-level behaviors.

**Conclusions:**

Determinants of quadruple therapy intensification vary by clinician specialty and care setting. Future research should explore implementation strategies that address these determinants by specialty and setting to promote health equity.

**Supplementary Information:**

The online version contains supplementary material available at 10.1007/s11606-025-09515-5.

## BACKGROUND

Heart failure (HF) is a condition with high morbidity and mortality that affects the physical and mental health of over 6.5 million US adults with a disproportionate impact on populations experiencing disadvantage.^1,2^ For the approximately 40% of the HF population with HF with reduced ejection fraction (HFrEF), randomized controlled trials have demonstrated the profound impact of medications from four drug classes at target dosing.^3^ Collectively referred to as foundational guideline-directed medical therapy (GDMT) or quadruple therapy, these medications improve quality of life, reduce the risk of hospitalization and mortality by 64%, and add an estimated eight life-years with comprehensive treatment with all four drug classes.^4,5^ Yet, data from US registries and health systems show consistently suboptimal use of these life-saving therapies.^6–12^

Determinants, or barriers and facilitators, of quadruple therapy intensification, defined as adding a new class of medication or increasing the dose on an already prescribed medication, have been examined using quantitative and qualitative approaches in primary care and cardiology practices in large health systems.^10,13–17^ Federally qualified health centers (FQHCs) are one of the main sites of primary care delivery in the USA, especially for patients, who are underinsured, uninsured, or have Medicaid. However, determinants of HF care in FQHCs have not been examined, and patients seen in this care setting are predominately from groups that are low-income, have the highest morbidity and mortality from HF, and have limited specialty care access.^18^ Due to the lack of scaling of previously tested strategies to increase GDMT prescription, better characterizing determinants across diverse settings will facilitate tailored implementation strategies by care setting and may contribute to increasing equitable outcomes for all patients with HF.

In this study, we sought to identify determinants of prescribing quadruple therapy from different specialties (primary care and cardiology) and care settings (large integrated health system and FQHCs) with a previously investigated conceptual model^19,20,21^ that integrates two widely used theories and frameworks from the social science and implementation science literature: the Theory of Planned Behavior (TPB) ^22^and the Consolidated Framework for Implementation Research (CFIR 2.0).^23^

## METHODS

### Study Design and Recruitment Procedures

We conducted a qualitative study with semi-structured interviews of clinicians. The study followed the Consolidated Criteria for Reporting Qualitative Research (COREQ) Checklist for reporting (Supplemental Table 1).^24^ The Northwestern University Institutional Review Board approved this study. All participants provided verbal informed consent.

Participants were recruited from a multi-county integrated health system and a multi-state FQHC network. Eligible participants included physicians, nurse practitioners, physician assistants, and pharmacists in cardiology and primary care. We used a purposive sampling approach.^25^ Details on recruitment procedures is provided in the Supplement.

### Data Collection

Data were collected during one-on-one, 30-min virtual interviews. The interviews were conducted using semi-structured interview guides tailored to clinician specialty. Information on reflexivity of key team members is included in the Supplement. Using CFIR, we developed questions to identify factors that impact the implementation of evidence-based care. We developed additional questions based on the Chronic Care Model, which includes specific elements of health systems associated with promoting high quality, evidence-based care.^26^ The interview guides and details on their development are included in the Supplement.

### Analysis and Theoretical Framework

Professionally transcribed interviews were analyzed in ATLAS.ti (Berlin, Germany)^27^ using thematic analysis, which reports themes identified in the data.^28^ Our analysis involved a hybrid inductive and deductive coding approach.^29^ We developed a set of codes a priori that were informed by the interview guide and literature. Two coders (LPG and SEP) independently coded 20% of the integrated health system and 20% of the FQHC transcripts and met to review coding discrepancies and the need for new or refined codes driven by the emergence of new themes. A detailed description of the coding process, including the role of thematic saturation, is included in the Supplement.

After coding was complete, FSA, LPG, and SEP independently prepared analytic memos, which allow team members to synthesize data into high-level themes.^30^ The memos addressed six questions (Supplemental Table 6) and were compared to identify common preliminary themes. In a post hoc, analytical decision, we elected to report findings in a previously investigated TPB-CFIR framework to better define the relationships between contextual multi-level determinants and clinician intention and action to intensify quadruple therapy.^19,20,21^ The TPB purports that intention is the most proximal contributor to behavior and is influenced by three constructs. The first construct, attitude, encapsulates the degree of favorability that an individual ascribes to a behavior. The second construct, subjective norms, references the social pressure surrounding behaviors. The third construct, perceived behavioral control, encompasses the perception of the ease or difficulty of performing the behavior. The lack of incorporation of multi-level determinants influencing beliefs and intentions represents a limitation of the TPB.^21^ The integration with CFIR 2.0, which comprehensively covers five multi-level ecological domains influencing implementation outcomes, provides a holistic picture of factors influencing quadruple therapy intensification.

## RESULTS

### Participant Demographics

We conducted a total of 31 interviews, including 23 health system and 8 FQHC clinicians (Table 1). The sample (*n* = 31) was predominantly female (61%) with a median age of 39 years (Supplement Table 7).

**Table 1:** Participant Clinical Specialties and Roles

	Integrated health system (*n* = 23)	FQHC (*n* = 8)
Specialty^a^, *n* (%)		
Cardiology	13 (57%)	0
Family Medicine	2 (9%)	6 (75%)
Internal Medicine	6 (26%)	2 (25%)
Role, *n* (%)		
Nurse practitioner	6 (26%)	1 (12%)
Pharmacist	4 (17%)	0
Physician	11 (48%)	5 (63%)
Physician assistant	2 (9%)	2 (25%)

^a^Two pharmacist participants were not affiliated with a specialty; thus, the specialty percentage total is < 100%

**Figure 1:**
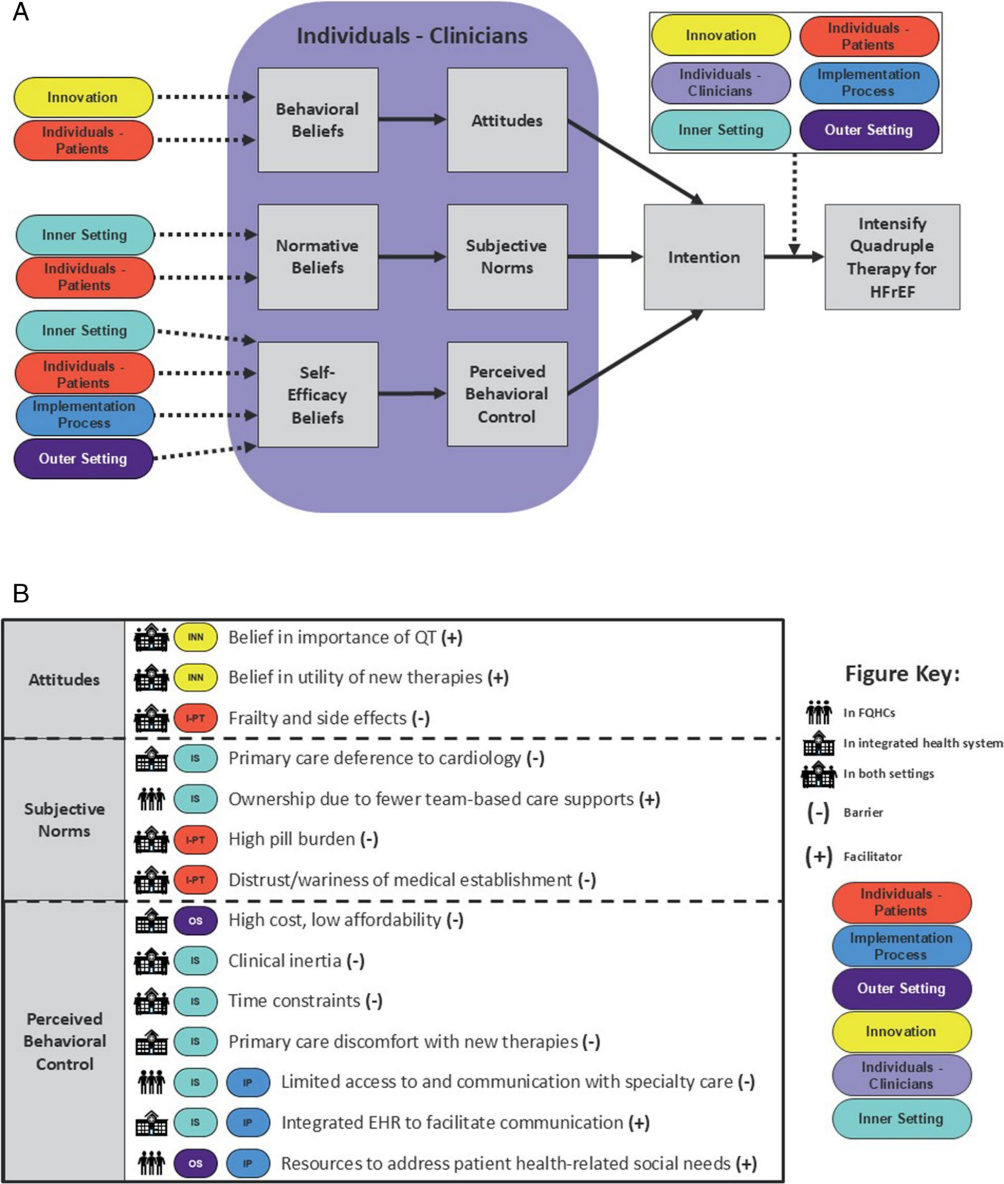
Applications of Theory of Planned Behavior and CFIR to a cardiology or primary care visit. **A** The relationship of CFIR domains, including innovation, individuals, inner and outer setting, and implementation processes on Theory of Planned Behavior constructs of attitude, subjective norms, and perceived behavioral control. The Theory of Planned Behavior constructs influence clinician intention and subsequent use of quadruple therapy for patients with HFrEF at the time of clinical visits in cardiology and primary care. The “Fig. 1 Key” lists the findings that map to the CFIR domains and TPB constructs. It also provides an overview of symbols that indicate if a determinant is a barrier or facilitator and if it is specific to the FQHC setting, integrated health system setting, or both. EHR, electronic health record; FQHC, federally qualified health center; HFrEF, heart faiure with reduced ejection fraction; QT, quadruple therapy.

A summary of thematic findings is presented in Fig. 1, which illustrates how CFIR domains inform TPB constructs. Table 8 in the Supplement lists determinants by setting type.

### CFIR Domains That Inform Attitudes

#### Innovation: The Thing Being Implemented (Intensification of Quadruple Therapy)

Participants described their attitude toward the prescription of quadruple therapy. As illustrated through the quotes in Table 2, there was unanimous support for the importance of quadruple therapy. Many participants’ perspective on their role in the prescription of quadruple therapy was influenced by their knowledge of the evidence base. Participants expressed that changes to the evidence base, such as the addition of new medications, were an important component of treatment. For example, participants noted that some of the medication classes, including Angiotensin-converting enzyme inhibitors (ACE) inhibitors, Angiotensin II receptor blockers (ARBs), and beta blockers, have served as the long-standing foundation of HFrEF treatment. Some participants noted that when Sodium-glucose cotransporter-2 inhibitors (SGLT2i) were added to the guidelines, they were less familiar with the medication class but have come to recognize the value of this medication class.

**Table 2:** Illustration of TPB and CFIR Constructs Through Participant Quotations

	Integrated health system–based participant	FQHC-based participant
TPB Construct: Attitudes CFIR Domain: Innovation	*It’s important because it helps them [patients] hopefully get a little bit better outcomes and helps them stay out of the hospital*	*It’s extremely important because we know that if they’re optimized under medical regimen, then they could avoid other interventions that are much more invasive*
TPB Construct: Subjective Norms CFIR Domain: Inner Setting	*I would say, 99 percent of the time, what I would do is, if the patient has a cardiologist, send them a message of saying, “Hey. What do you think about an ARNI in this patient?” Then leave it up to them to decide whether to prescribe it*	*I have patients from all over the world, and I think that there are certain communities where the provider is the end all be all. There are other communities where medicines are great. The more medicines you prescribe the better, and there are other communities where it’s like, “I feel fine. I’m not gonna do this”*
TPB Construct: Perceived Behavioral Control CFIR Domain: Inner Setting	*With heart failure physicians, this is what your primary job is. What you’re in-tuned to doing or attuned to doing every day. But when I get referrals for new patients, I would say that clinician inertia is a big part of the problem for sure Because the patient, “feels okay”, but will be on a non-guideline directed beta blocker*	*My patients may be dealing with other things that are much more pressing, frankly. Good examples would be substance use disorder. There’s a lot of fatalities from that. I have a lot of patients who have opioid use disorder. That’s much more quickly going to kill them than their heart failure*
TPB Construct: Perceived Behavioral Control CFIR Domain: Outer Setting	*I think it’s very difficult to know what the [insurance] coverage is. Especially for ARBs and the SGL2 inhibitors. Usually we end up sending it to the pharmacy. Then, calling the pharmacy and doing a price check. If we could price check it before sending it over. That we could tell the patient what they could expect in terms of cost*	*If they do qualify, because of their income, then we can also use the patient assistance program which we often do, particularly for the ARNIs and the SGLT2 s because they’re pretty expensive as well, so we’ll use the patient medication assistance program to cover their medications*
TPB Construct: Perceived Behavioral Control CFIR Domain: Implementation Process	*I always send notes or messages through the EHR. And it’s gone pretty well. They’re very responsive from the physicians. Or if they can’t respond then their nurse or somebody responds*	*Unfortunately, I think that’s a fragmented issue at least in our healthcare system because we are not necessarily affiliated with any tertiary hospital center. It requires me to be on top of the patient’s care. A lot of times I’m unaware of the fact that they went to see the cardiologist or that the cardiologist changed their medications*

#### Individuals-Patients: Patient-Related Factors That Impact Quadruple Therapy Intensification

Patients are the recipients of the innovation, quadruple therapy. There are some recipient characteristics, which impact experience with quadruple therapy. Participants described challenges associated with quadruple therapy–related decision-making for frail patients. Several participants noted that side effects, such as hypotension, may be especially problematic for these patients. In some instances, side effects informed participants’ attitude toward initiating or adjusting quadruple therapy.

### CFIR Domains That Inform Subjective Norms

#### Inner Setting: Factors Related to the Site That Impact Quadruple Therapy Intensification

Participants described the impact of subjective norms and the inner setting of their clinical environment on their decision to prescribe or adjust quadruple therapy. In contrast to cardiology participants and as illustrated by the quote from an integrated health system participant in Table 2, primary care participants reported variable comfort with prescribing and increasing the dose of Angiotensin receptor/neprilysin inhibitors (ARNIs). A clinician’s understanding of the division of responsibility between primary care and cardiology and in the management of different diagnoses may influence their willingness to take ownership of HF management. Among participants, this phenomenon was most apparent in primary care clinicians’ deference to cardiologists regarding the management of HFrEF medications. Even in instances where primary care clinicians noted that they were willing to adjust HF medications, many noted the importance of consulting with the cardiologist. Participants from the integrated health system reflected on a collaborative approach to care that included physicians, advanced care practitioners, and pharmacists. Alternatively, FQHC-based participants observed that this team-based care approach for HFrEF was less common in their practices with more responsibility falling to primary care clinicians.

#### Individuals-Patients: Patient-Related Factors That Impact Quadruple Therapy Intensification

When speaking to patient knowledge, many participants noted that their patients do not always understand the importance of quadruple therapy and grow frustrated by pill burden, especially when taking medications for multiple conditions. Another common patient-related need involved addressing wariness of the medical establishment, which sometimes stemmed from information that patients heard in television commercials. Others reflected that their patients were hesitant about the use of quadruple therapy because they preferred nonpharmaceutical remedies, including dietary changes. While these patient-level determinants present barriers to the prescription of quadruple therapy, many clinician participants highlighted the potential role of patient education as a mechanism to achieve target medication doses. Participants offered examples of patient education initiatives, including animations and teach back techniques, that can help patients understand the diagnosis and roles of medication classes in symptom management.

### CFIR Domains That Inform Perceived Behavioral Control

#### Individuals-Patients: Factors Related to Patients That Impact Quadruple Therapy Intensification

The most frequently mentioned patient-related factors impacting perceived behavioral control were cost and affordability. Participants noted that the cost of specific medications, including ARNIs and SGLT2i, may be particularly burdensome. FQHC-based participants also reflected on the role that patient population characteristics played in their decision to initiate and titrate HF medications. While integrated health system–based participants described the challenges that comorbidities present in the management of HF medications, FQHC-based participants highlighted the challenges presented by specific comorbidities, such as substance use disorders and the impacts of social determinants of health, including housing insecurity. FQHC-based participants more frequently mentioned the impact of patients’ health literacy and fewer touchpoints with the health system.

#### Inner Setting: Factors Related to the Site of Implementation of Quadruple Therapy

Participants identified several factors in the inner setting that influence behavioral control and may facilitate or hinder the prescription of quadruple therapy. First, some primary care participants described the impact of clinical inertia, noting that they may be hesitant to change a medication dose if the current dose was well-tolerated. In contrast, cardiology participants, such as the one quoted in Table 2, described less hesitancy about initiating new medications or up-titrating existing medications when patients are doing well. In fact, they were focused on continuing to make changes to adhere to guidelines. Time constraints may also hinder a clinician’s ability to optimize quadruple therapy and impact the innovation’s compatibility with the inner setting. Some participants described factors that may aid in surmounting time constraints, including advanced practice clinician-facilitated communication between primary care and cardiology to discuss potential changes to the HF medications. Participants noted that access to effective communication mechanisms between cardiology and primary care may also impact the prescription of quadruple therapy. While participants in the integrated health system described the benefits of an electronic health record (EHR) system that facilitated communication among clinicians and between patients and clinicians, FQHC-based participants noted the challenges they faced communicating with cardiology specialists, who were outside of their health system.

#### Outer Setting: Factors at the Setting in Which the Inner Setting Exists

When the issue of cost and affordability was discussed, there was some indication, as illustrated by the quote in Table 2, from FQHC-based participants that existing programs for their patients help overcome these barriers, which speaks to the role that local conditions play in supporting the implementation of quadruple therapy. More specifically, many of their patients are on Medicaid and qualify for income-based patient assistance programs that help to reduce medication costs. Other participants described the role of organizational-level programs, such as the 340B Program, which allow participating organizations to procure outpatient medications at a discount. While these programs offer avenues to address affordability challenges, some participants noted that challenges persist, especially surrounding insurance coverage of SGLT2i and ARNIs.

#### Implementation Process: Activities and Strategies Used to Implement Quadruple Therapy

A comparison of findings from the participants from integrated health systems vs. FQHCs indicates some differences in their experiences with prescribing quadruple therapy. As described above, FQHC-based primary care clinicians often indicated that they were more willing to initiate and titrate HF medications compared to primary care clinicians based at an integrated health system due to inconsistent and fragmented communication with cardiology specialists. Fragmented communication can contribute to a greater burden on the primary care clinicians to determine what changes have been made, and this process can be further complicated by patients’ language barriers.

## DISCUSSION

Using the combined TPB and CFIR 2.0 conceptual model, we highlight multi-level determinants that inform the action of quadruple therapy intensification and illustrate how these determinants influence the action of quadruple therapy intensification.^19–21^ We also identified differences between clinician types and between those from different types of organizations regarding their comfort initiating and titrating HF medications. Primary care clinicians were more likely to describe clinical inertia as a barrier to quadruple therapy intensification compared to cardiology clinicians. Among primary care clinicians, those at FQHCs were typically more comfortable managing quadruple therapy compared to their integrated health system counterparts, who often deferred to cardiology clinicians to manage the regimen. Within the integrated health system, co-management was facilitated through EHR-based communication while primary care clinicians based at FQHCs struggled to co-manage patients with cardiology specialists due to lack of integrated EHR and patients’ lack of established relationships with specialists. Both integrated health system and FQHC clinicians identified patient-related factors that complicate HF medication management, including frailty, side effects, and pill burden. FQHC clinicians also highlighted additional patient-related barriers, including health-related social risks like housing insecurity and complex comorbidities including substance use and mood disorders.

This study extends the growing literature on barriers and facilitators to prescription of evidence-based therapies in HF, including clinical inertia.^13–15,31–36^ Our findings are complementary to a taxonomy that accounts for the role of clinical inertia in treatment non-intensification that was applied to HFrEF trial data and adds a conceptual model that identifies potential causal mechanisms between multi-level determinants and quadruple therapy intensification.^32^ One qualitative study of cardiology and primary care clinicians at a different integrated health system identified similar challenges related to clinical inertia, pill burden, competing priorities, affordability of SGLT2is and ARNIs, and diffusion of responsibility for care across the health system.^13,16,17^ Clinical inertia has also been documented across a range of chronic conditions.^37–39^ Strategies to address clinical inertia have been tested in HF and many other conditions. Implementation strategies, such as EHR-based alerts, patient activation tools, and multicomponent interventions, have been tested to overcome clinical inertia and other barriers to improve the uptake of quadruple therapy,^11,32,45–48^ Similarly, in the management of hypertension, multiple strategies—including ambulatory pressure monitoring, physician reminders, and educational interventions for primary care clinicians—have been associated with improvements in blood pressure control.^38^

Our study provides more insight into the role that EHR-based communication between primary care and cardiology clinicians can play in facilitating the management of quadruple therapy and adds a comparison with FQHCs. These findings align with other published literature on the benefits of EHR interoperability including reductions in costs and adverse patient safety events.^40^ Our findings are also complementary to those from a study that assessed the perspectives of primary care physicians on their experience co-managing patients with chronic kidney disease with nephrologists and identified barriers related to timely information exchange, unclear roles and responsibilities, and limited access to nephrologists.^41^

As illustrated in Fig. 1, there are determinants within FQHCs at the level of the inner setting that have implications on the optimization of quadruple therapy. Challenges include limited access to specialty care and distrust of the medical establishment, both of which may be exacerbated by time constraints and competing priorities. While these challenges are not insignificant, there may be opportunities to leverage available resources that address patient health-related social needs. Existing research on the implementation of quadruple therapy within FQHCs is limited, but our findings can be compared to the treatment of mental health diagnoses in primary care. Prior qualitative research that assessed clinician perspectives found that competing priorities, such as patients with multiple health concerns, and discomfort managing psychotropic medications can complicate the management of mental health diagnoses by primary care clinicians.^42^ Other research in this space has demonstrated that FQHC-based clinicians face communication challenges when referring patients to more specialized mental health treatment.^43^ This is similar to findings from our FQHC-based participants, who indicated that they have difficulty referring their patients to cardiology specialists and encounter communication challenges due to lack of integrated EHRs. Finally, interprofessional collaborative care for patients with multiple chronic conditions, including depression and high cholesterol, has been suggested as a mechanism to better serve patients who receive care in FQHCs.^44^ A similar approach might be beneficial for patients with HFrEF in FQHCs given the potential for cross-specialty collaboration in the management of quadruple therapy.

Quadruple therapy represents an evidence-based practice that can enhance cardiovascular health equity by improving patient outcomes across diverse populations.^35^ Although some implementation strategies have been led to improvements in the uptake of quadruple therapy,^11,32,45–48^ none, to our knowledge, has prioritized reaching patients who primarily receive care in FQHCs. Moreover, implementation studies for quadruple therapy have prioritized the evaluation of process and clinical outcomes, but largely have not evaluated costs or implementation strategy mechanisms.^35,49,50^ Future research should test tailored strategies based on determinants and settings, employ a comprehensive evaluative framework with extensions to promote health equity,^51,52^ and measure costs. A comprehensive evaluation will increase the likelihood of scaling and sustaining successful implementation strategies.

This study has several limitations and strengths. We had disparate sample sizes from each setting type, which may introduce selection bias. However, this study, to our knowledge, provides the first qualitative assessment of FQHC clinician perspectives on determinants of quadruple therapy for patients with HFrEF. Physicians represented 52% (*n* = 16) of our total sample size. Considering the interdisciplinary nature of care for patients with HF, future research should continue to explore the perspectives of other members of the care team, including pharmacists and advanced care practitioners. Our study identified clinician-identified, patient-level determinants because interviewing patients directly from each setting was outside the scope of the goals and resources of the study which sought to identify determinants of the prescription of quadruple therapy. Future work will identify strategies targeting determinants of prescription of quadruple therapy from the patient perspective across diverse settings, including FQHCs. While the generalizability of qualitative findings is limited, the findings may be transferable to other contexts, but the applicability will depend on the similarity of patient populations and care delivery setting. We also acknowledge there is the potential for overlap with some of the CFIR domains. For instance, patient assistance programs may stem from policies in the outer setting but their success is influenced by factors within the inner setting.

## CONCLUSION

Key barriers to the uptake of quadruple therapy include clinician comfort with medication adjustment, understanding division of responsibility within a collaborative care team, communication challenges between clinicians from different specialties, side effects, and comorbidities. The need to identify implementation strategies to improve the uptake of quadruple therapy that address barriers at the patient-, clinician-, and organizational-level persists. Based on the number of barriers aligned with the CFIR domain of inner setting, we especially recommend future research that explores strategies to address organizational-level barriers. Addressing barriers at this level has the potential to improve the experience that clinicians have with quadruple therapy as deliverers of the intervention and patients as the recipients. Such research can consider the needs of primary and cardiology clinicians and may also help to address some of the unique challenges faced by community-based care settings.

## Supplementary Information

Below is the link to the electronic supplementary material.

Supplementary file1 (DOCX 74.8 KB)

## Data Availability

Due to the sensitive nature of qualitative data and because participants did not provide consent for the sharing of their full interview transcripts, the data supporting the findings of this study are not publicly available. The interview guides used in the study are available in the Supplement.

## Abbreviations

HFrEF: Heart failure with reduced ejection fraction
GDMT: Guideline-directed medical therapy
FQHC: Federally qualified health centers
TPB: Theory of Planned Behavior
CFIR 2.0: Consolidated Framework for Implementation Research 2.0
COREQ: Consolidated Criteria for Reporting Qualitative Research
ACE inhibitors: Angiotensin-converting enzyme inhibitors
ARBs: Angiotensin II receptor blockers
SGLT2i: Sodium-glucose cotransporter-2 inhibitors
ARNIs: Angiotensin receptor/neprilysin inhibitors
EHR: Electronic health record
